# Patient satisfaction with clinical laboratory services and associated factors among adult patients attending outpatient departments at Debre Markos referral hospital, Northwest Ethiopia

**DOI:** 10.1186/s13104-019-4558-8

**Published:** 2019-08-19

**Authors:** Asmamaw Alelign, Yihalem Abebe Belay

**Affiliations:** 1 Debre Markos Referral Hospital, Debre Markos, Ethiopia; 2 Department of Public Health, Debre Markos University, Debre Markos, Ethiopia

**Keywords:** Debre Markos hospital, Patient satisfaction, Laboratory services

## Abstract

**Objectives:**

There is limited evidence on patient satisfaction with clinical laboratory services in Northwest Ethiopia. This study aimed to assess patient satisfaction with clinical laboratory services and associated factors among adult patients attending outpatient departments at Debre Markos hospital, Northwest Ethiopia. Cross-sectional study was conducted among 391 patients from April 09–16, 2019 using interviewer-administered questionnaire. Systematic random sampling technique was used. Data were entered into Epi-Data software version 4.2, and analyzed using SPSS 23. Binary logistic regression analysis was employed. Variables having *p*-value < 0.05 were considered as statistically significant.

**Results:**

Overall level of patient satisfaction towards clinical laboratory services was 48.3%. Patients with no occasion of missing laboratory results [AOR: 2.10; 95% CI (1.13, 3.83)], had diplomas and above educational level [AOR: 0.38; 95% CI (0.15, 0.96)], and who reported no place in the laboratory to put personal things [AOR: 0.37; 95% CI (0.21, 0.54)] were significant factors. Hospital administration and laboratory department in Debre Markos hospital should strengthen their effort to improve patient satisfaction focusing on educational status of patients, missing laboratory results, and the availability of places to put personal things in the blood drawing room.

## Introduction

Patient satisfaction is a crucial and commonly used indicator for measuring the quality in any health care system. Patient satisfaction has positive effect on clinical improvement, patient adherence and retention, job satisfaction and appropriate clinical care by physicians [1–3]. On the other hand, mismatch between patient expectation and the service they received leads dissatisfaction. Patient satisfaction towards clinical laboratory service is influenced by the quality of service and professionalism of the staff, provision of adequate information to collect specimen and when and how to receive laboratory results, waiting time to receive laboratory results, availability of ordered laboratory tests, cleanness of the laboratory room, location of laboratory room, availability and accessibility of latrine [4–9].

Patient’s satisfaction on laboratory services has not yet exhaustively studied in Ethiopia. Thus, this study aimed to investigate patient satisfaction with clinical laboratory services and associated factors among adult patients attending outpatient departments at Debre Markos referral hospital, Northwest Ethiopia.

## Main text

### Methods

#### Study design, area and period

An institution based cross-sectional study was conducted from April 09 to 16, 2019 at Debre Markos Referral Hospital. The average number of patients monthly treated in the hospital as an outpatient was 5658 from the three wards internal medicine (2100), surgery (1800) and Gynecology/Obstetrics (1758) respectively [10].

#### Population and eligibility criteria

The source population was all adult patients (≥ 18 years) visiting laboratory service from internal medicine, surgery, Gynecology/Obstetrics departments at Debre Markos Referral Hospital. The study population was all adult patients (≥ 18 years) visiting laboratory service from internal medicine, surgery and Gynecology/Obstetrics departments at Debre Markos Referral Hospital during the data collection period. All adult patients at the internal medicine, surgery and Gynecology/Obstetrics outpatient departments who were requested for clinical chemistry, hematology, parasitological and urine analysis tests were included in the study. However, patients who had mental illness and hearing impairments were excluded from the study.

#### Sample size determination and sampling procedure

The required sample size was calculated using a single population proportion formula;

$$ {\text{n}} = \frac{{\left( {{{\text{z}\upalpha } \mathord{\left/ {\vphantom {{z\alpha } 2}} \right. \kern-0pt} 2}} \right)^{2} \;{\text{p}}\;\left( {1 - {\text{p}}} \right)}}{{{\text{d}}^{2} }} $$

#### Assumptions

n = required sample size, Z = critical value for normal distribution at 95% confidence level (1.96), d = 0.05 (5% margin of error), P = 63.3% (proportion of patients satisfied with laboratory service) [11] and an estimated non-response rate of 10%. The final calculated sample size for this study was 391. Systematic random sampling technique was used to select study participants with k-values 3, 4, 4 respectively. Proportional allocation to population size (170 from Internal medicine, 112 from Surgery, and 109 from Gynecology/Obstetrics outpatient departments) was employed.

#### Data collection procedure

Data were collected using a pre-tested and structured interviewer administered questionnaire. The questionnaire was prepared in English and translated to Amharic, then back to English to check for its consistency. The questionnaire contains satisfaction indicators which were related to socio-demographic characteristics of the patients and different dimensions of laboratory services such as waiting time (turnaround time), availability of requested laboratory tests, convenience of service hours, and type of laboratory visit, privacy, respect, courtesy and confidentiality. The study participants were asked to rate each aspects of the laboratory service on five point liker scale (1 = Very Dissatisfied, 2 = Dissatisfied, 3 = Neutral, 4 = Satisfied, 5 = Very satisfied). Exit interview of patients was carried out.

To assure the data quality, two diploma laboratory technicians and one BSc laboratory professional were recruited as data collectors and supervisor, respectively. In addition, training regarding the study objectives and data collection process was given for data collectors and supervisor for 2 days. Moreover, the questionnaire was pretested among 5% of the sample size at Finoteselam hospital. Furthermore, intensive supervision was done by supervisor and principal investigators throughout the data collection period.

#### Study variables

The dependent variable of this study was patient satisfaction. Patient satisfaction with laboratory services was defined as the patient’s opinion of the care received from the institution and is acknowledged as an outcome indicator of the quality of health care. i.e. ≥ mean value = Satisfied and < mean value = Dissatisfied [7].

The independent variables were: Socio-demographic characteristics (age, gender, level of education, marital status, occupation, income and residence), waiting time to get service, availability of requested lab tests, convenience of service hours, confidentiality of lab results, improvement of service from time to time, queue process to get service, availability of service providers at their job, missing results, cost of laboratory service, location of laboratory, hospitality of laboratory professionals to patient, cleanness of latrine, needle stick attempted, and availability of equipment.

#### Data processing and analysis

Data were cleaned, coded and entered using Epi-Data software Version 4.2 and analyzed using SPSS Version 23. Binary logistic regression was employed. In the bivariable analysis, variables with p-value < 0.25 were fitted into the multivariable model. Finally, adjusted odds ratios with their 95% confidence intervals were estimated to assess the strength of association, and variables with p-value < 0.05 were considered statistically significant factors.

### Results

#### Socio-demographic characteristics of the study participants

A total of 391 laboratory service users were included in the study, resulting in a response rate of 100%. About 244 (57.3%), and more than two-third (68.7%) of the respondents were females and married respectively. The mean age of participants was 36.9 (± 14.08) years with a range of 18–75 years. Nearly half of the respondents were unable to read and write (47.8%) and almost one-third (30.4%) of them were housewives. Majority (86.2%) had reported that they earn an average family monthly income of more than 500 Ethiopian Birr. Almost half of the participants resided in rural areas (Table 1).

**Table 1:** Socio-demographic characteristics of hospital laboratory service users at Debre Markos referral hospital, Northwest Ethiopia, 2019 (n = 391)

Variables	Category	Frequency	Percentage
Sex	Male	167	42.7
	Female	224	57.3
Age in years	18–27	123	31.4
	28–37	114	29.2
	38–47	57	14.6
	48–57	54	13.8
	≥ 58	43	11.0
Educational status	Unable to read and write	187	47.8
	Able to read and write	31	7.9
	Grade 1–8	53	13.6
	Grade 9–12	53	9.0
	Diploma and above	85	21.7
Marital status	Married	268	68.6
	Single	70	17.8
	Divorced	25	6.4
	Widowed	28	7.2
Occupation	Student	70	17.9
	Housewife	119	30.4
	No employment	20	5.2
	Merchant	48	12.3
	Government worker	33	8.4
	Farmer	101	25.8
Average family monthly income (in Ethiopian Birr)	≤ 500	54	13.8
	> 500	337	86.2
Residence	Rural	201	51.4
	Urban	190	48.6

#### General service parameters and patients’ satisfaction towards clinical laboratory services

The overall patient satisfaction on laboratory services was determined by taking mean score and above for 16 variables that were utilized to reflect satisfaction. Nearly half (48.3%) of patients were satisfied with the general clinical laboratory services provided. Majority of the study participants (89.5%) had got the service with payment. Majority (83.4%) of the respondents have got all the requested laboratory tests that the clinicians ordered. Nearly half (44%) of the patients were first time service users at the hospital laboratory. When the respondents were asked if they come again to get services at the hospital laboratory, almost all (96.2%) of them were interested to come again. Among the reason to come again the same hospital laboratory to get service were: good quality of service (26.3%), preferred service providers (21.3%), availability of many services (21.0%), cheap service fee (12.3%), get service early (9.7%) and convenience of working hour (4.6%). In contrary to the previous, the reasons for not coming again to the same hospital to get laboratory services were long waiting time. Regarding the turnaround time to get results, the majority (82.4%) responded that they wait more than 1 h and nearly two-third (67%) of participants had one vein puncture attempt (Table 2).

**Table 2:** General service parameters of Hospital laboratory service users at Debre Markos referral hospital, Northwest Ethiopia, 2019 (n = 391)

Variable	Frequency	Percent (%)
Service payment		
Payment	350	89.5
Free	41	10.5
Do you know this laboratory before?		
Yes	219	56.0
No	172	44.0
Improvement of service (n = 219)		
Yes	145	66
No	74	34
Turnaround time		
< 30 min	17	4.3
30 min–1 h	52	13.3
> 1 h	322	82.4
Will you come again to the hospital laboratory		
Yes	376	96.2
No	15	3.8
Reason to decide to come again		
Convenient opening hour	18	4.6
Get the service early	38	9.7
Good quality service	103	26.4
Availability of many services	82	21.0
Cheaper service fee	48	12.3
Preferred service provider here	83	21.2
Others	4	1.0
Reason to decide not to come again (n = 15) long waiting time	15	3.8
Needle stick attempted to draw blood		
One vein puncture	262	67.0
Two vein puncture	88	22.5
Three vein puncture	30	7.7
Four or more vein puncture	11	2.8
Place in blood drawing room to put personal thing		
Yes	148	37.9
No	243	62.1
Occasion of missing results		
Yes	65	16.6
No	326	83.4
Information given on how to decrease possible bruise		
Yes	245	62.7
No	146	37.3

#### Factors affecting the level of patients’ satisfaction towards clinical laboratory services

In the bivariable analysis, educational status of respondents, occupation, residence, occasions of missing laboratory results, information to decrease possible bruise, place to put personal thing, convenience of opening hour were found to be associated with patient satisfaction on clinical laboratory services.

These factors were further analyzed using multivariable logistic regression by enter method. The results of multivariable logistic regression analysis showed that the educational level, missing of laboratory results, and availability of place in blood drawing room to put personal things were found to have a statistically significant association with the overall satisfaction of patients toward clinical laboratory services.

The likelihood of patient satisfaction on clinical laboratory services was 2.1 times more likely in patients who had no occasion of missing laboratory results as compared with who had occasion of missing results (AOR = 2.10, 95% CI 1.13, 3.83).

On the other hand, patients who were able to read and write and who had diplomas and above educational level were less likely to be satisfied with the laboratory services compared to those who were not able to read and write (AOR = 0.41, 95% CI 0.18, 0.92 and AOR = 0.38, 95% CI 0.15, 0.96) respectively. Similarly, patients who said no place in the laboratory to put personal things were less likely to be satisfied with the laboratory service compared to their counterparts (AOR = 0.34, 95% CI 0.21, 0.54) (Table 3).

**Table 3:** Factors associated with patient satisfaction towards clinical laboratory service among adult patients attending outpatient departments at Debre Markos referral hospital, 2019 (n = 391)

Variable	Satisfaction		Crude OR (95% CI)	Adjusted OR (95% CI)
	Satisfaction n (%)	Dissatisfaction n (%)		
Education				
Unable to read and write	79 (20.25)	108 (27.6%)	1.00	1.00
Able to read and write	13 (3.3%)	18 (4.6%)	0.30 (0.19, 0.56)	0.41 (0.18, 0.92)
Grade 1–8	23 (5.9%)	30 (7.7%)	0.30 (0.14, 0.74)	0.52 (0.14, 3.26)
Grade 9–12	15 (3.8%)	20 (5.1%)	0.34 (0.17, 0.67)	0.47 (0.20, 1.11)
Diploma and above	59 (15.1%)	26 (6.6%)	0.33 (0.15, 4.10)	0.38 (0.15, 0.96)
Place to put personnel things				
Yes	52 (13.3%)	96 (24.6%)	1.00	1.00
No	137 (35.0%)	106 (27.1%)	0.42 (0.28, 0.64)	0.34 (0.21, 0.54)
Occasion of missing results				
Yes	42 (10.7%)	23 (5.9%)	1.00	1.00
No	147 (45.8%)	179 (37.6%)	2.2 (1.30, 3.90)	2.10 (1.13, 3.83)

### Discussion

Patient centered care is one of the six domains of health care quality developed by World Health Organization. The aim of this study was to examine the satisfaction of patients with clinical laboratory services and its associated factors among adult patients attending outpatient departments at Debre Markos referral hospital, Northwest Ethiopia.

The overall client satisfaction towards medical laboratory services in study area was 48.3%. This is in line with the findings of the study at Amhara Region, Northwest Ethiopia, 52.6% [12]. The possible explanation for the low satisfaction rate might be due to excess flow of patients to the laboratory as Debre Markos referral hospital is the only referral hospital used for East Gojjam and West Gojjam, Awi, West Wollega and South Wollo Zones. But, the current finding is slightly lower than a report from a study conducted in a teaching hospital in Addis Ababa (59.7%) [4] and Nekemte Hospital, Oromia Region (60.4%) [13]. The other studies from Eastern Ethiopia (87.6%) [8], and Southern Ethiopia (90.8%) [14] are much higher than the current study. This might be attributed to a difference in study area, period and patient flow.

The findings of this study showed that patients who were able to read and write and who had diplomas and above educational level were less likely to be satisfied with the laboratory service compared to those who cannot able to read and write, which is supported by similar study conducted in Tikur Anbesa Specialized Hospital, Addis Ababa [4] and Sidama Zone, Southern Ethiopia [14]. This might occur due to the fact that patients who were educated expect more professionalism from service providers and also quality service and infrastructures.

Those patients who reported no place available in the laboratory room to put personal things were less likely to be satisfied with the laboratory service compared their counterparts. It is comparable with a study conducted in Tikur Anbesa Specialized Hospital patients [4]. The absence of place in the laboratory room might lead patients to think about their possession decreasing their satisfaction on the service they received.

The odds of patients who had no occasion of missing laboratory results were 2.1 times more likely to be satisfied with the service than those who had occasion of missing results. This may be due to patients were susceptible to unethical additional service fee and long waiting time to get results with associated delay in getting the clinical health provider service. But, there was no similar significant association in other similar studies the researchers tried to search for even though the percentage of patients who complains missing laboratory results was 16.6% in this study, which is nearly equivalent to a similar study done in Nigeria (17.9%) [15].

In conclusion, the overall patients’ satisfaction towards clinical laboratory services was low. The educational level, missing of laboratory results, and availability of place in blood drawing room to put personal things were found to have a statistically significant association with the overall satisfaction of patients towards clinical laboratory services. The hospital administration and laboratory department in Debre Markos referral hospital should strengthen their effort to improve patient satisfaction focusing on educational status of patients, missing laboratory results, and the availability of places to put personal things in the blood drawing room. Proper service availability and readiness plan should be prepared and implemented to satisfy patients towards the laboratory services rendered at Debre Markos referral hospital.

## Limitations of the study

This study has the following limitations: the evidence of the study would have been enriched if it was supported by qualitative method. The perspectives of clinicians, service providers, and patients admitted in the hospital were not included. Face-to-face interview of respondents at the hospital might have exposed the study for social desirability bias.

## Data Availability

All data generated or analyzed during this study are included in this published article and its additional information files.

## Abbreviations

SPSS: statistical package for social sciences
CI: confidence interval
COR: crude odds ratio
AOR: adjusted odds ratio
